# ATF3 functions as a novel tumor suppressor with prognostic significance in esophageal squamous cell carcinoma

**DOI:** 10.18632/oncotarget.2322

**Published:** 2014-08-13

**Authors:** Jian-Jun Xie, Yang-Min Xie, Bo Chen, Feng Pan, Jin-Cheng Guo, Qing Zhao, Jin-Hui Shen, Zhi-Yong Wu, Jian-Yi Wu, Li-Yan Xu, En-Min Li

**Affiliations:** ^1^ Key Laboratory of Molecular Biology in High Cancer Incidence Coastal Chaoshan Area of Guangdong Higher Education Institutes, Medical College of Shantou University, Shantou 515041, P. R. China; ^2^ Department of Biochemistry and Molecular Biology, Medical College of Shantou University, Shantou 515041, P. R. China; ^3^ Department of Experimental Animal Center, Medical College of Shantou University, Shantou 515041, P. R. China; ^4^ Institute of Oncologic Pathology, Medical College of Shantou University, Shantou 515041, P. R. China; ^5^ Department of Pathology, Shantou Central Hospital, Affiliated Shantou Hospital of Sun Yat-Sen University, Shantou 515041, P. R. China; ^6^ Department of Oncologic Surgery, Shantou Central Hospital, Affiliated Shantou Hospital of Sun Yat-Sen University, Shantou 515041, P. R. China

**Keywords:** ATF3, independent prognostic factor, cell invasion and metastasis, MMP-2, esophageal squamous cell carcinoma

## Abstract

ATF3 was a transcription factor involved in the progression of certain cancers. Here, we sought to explore the expression and biological function of ATF3 in esophageal squamous cell carcinomas (ESCC). The prognostic significance of ATF3 expression was evaluated in 150 ESCC samples and 21 normal squamous cell epithelium tissues. Results showed that ATF3 was down-regulated in ESCC lesions compared with paired non-cancerous tissues and low tumorous ATF3 expression significantly correlated with shorter overall survival (OS) and disease-free survival (DFS). Cox regression analysis confirmed that ATF3 expression was an independent prognostic factor. Experimentally, forced expression of ATF3 led to decreased growth and invasion properties of ESCC cells *in vitro* and *in vivo*, whereas knockdown of ATF3 did the opposite. Furthermore, ATF3 upregulated the expression of MDM2 by increasing the nuclear translocation of P53 and formed an ATF3/MDM2/MMP-2 complex that facilitated MMP-2 degradation, which subsequently led to inhibition of cell invasion. Finally, we showed that Cisplatin could restrain the invasion of ESCC cells by inducing the expression of ATF3 via P53 signaling. Combined, our findings highlight a suppressed role for ATF3 in ESCC and targeting ATF3 might be a potential therapeutic strategy.

## INTRODUCTION

Esophageal squamous cell carcinoma (ESCC) is one of the most fatal malignancies worldwide. It is difficult to diagnose ESCC at early stages of disease development, and advanced ESCC frequently presents with extensive local invasion or regional lymph node metastasis [1]. The overall 5-year survival rate remains below 40% [2–4]. Metastatic tumors are often refractory or only partially sensitive to current therapeutic strategies and the primary cause of cancer related mortality [5]. Identification of molecular markers associated with the progression of this disease and understanding the key pathogenic processes involved remain crucial for improving outcome.

Activating transcription factor 3 (ATF3) is a member of the ATF/CREB family of transcription factors [6]. It is an adaptive-response gene that participates in cellular processes to adapt to extra- and/or intracellular changes, where it transduces signals from various receptors to activate or repress gene expression. ATF3 has been shown to dimerise with other ATF/CREB proteins, including ATF2, c-Jun, Jun B, and Jun D. Depending on the promoter context, these heterodimers can act as either repressors or activators of transcription. Therefore, the role of ATF3 as a repressor or activator of transcription cannot be generalized [7].

Recently, more and more evidence indicates that ATF3 may play a critical role in the progression of cancer. Interestingly, ATF3 has been demonstrated to play differing roles in cancer development depending on the cell type and context. ATF3 expression was found to be elevated in several cancers such as breast cancer and Hodgkin lymphomas [8, 9]. ATF3 may be oncogenic as it can be protective against apoptosis and, in many cases, can also promote metastasis of cell lines *in vitro* and *in vivo* [7]. In contrast to these, ATF3 was found to be induced following DNA damage in HCT-116 and RKO colon carcinoma cells and suppressed the growth of HeLa cells [10]. Over-expression of ATF3 reduced the invasive potential of ovarian cancer cells, bladder cancer cells and lung cancer cells [11–13]. Moreover, ATF3 can be induced by a range of anti-tumorigenic compounds, including curcumin, non-steroidal anti-inflammatory drugs, and the phosphatidylinositol inhibitor, LY294002 [14–16]. All these findings strongly suggest that ATF3 may be a novel therapeutic target.

The expression pattern and possible function of ATF3 in ESCC are still unclear. In the present study, we sought to determine the role of ATF3 expression in ESCC pathogenesis and the underlying molecular mechanisms. We discovered a novel ATF3/MDM2/MMP-2 complex, which was altered in ESCC and critically regulated ESCC progression and metastasis.

## RESULTS

### Reduced ATF3 expression in ESCC versus non-cancer tissues

**Figure 1:**
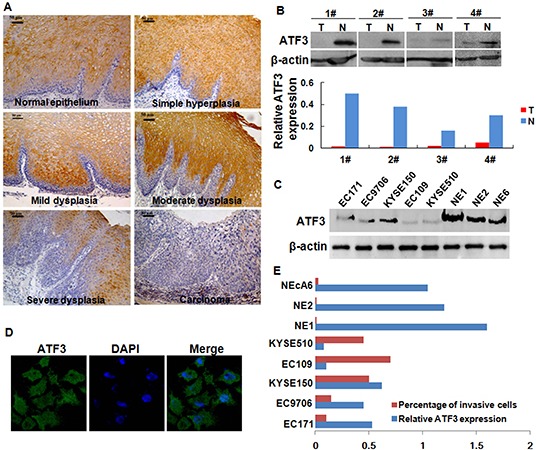
Expression of ATF3 in ESCC tissues and ESCC cell lines **(A)** Expression of ATF3 in the progression from normal epithelium to carcinoma of esophagus. Scale bar, 50μm. **(B)** Expression of ATF3 protein in four randomly selected, paired ESCC samples and matched normal tissues was analyzed by Western blotting. Signal intensity for the expression of ATF3 was quantified by densitometric scanning and normalized by internal control (β-actin). **(C)** ATF3 levels in whole-cell extracts were determined in various ESCC cell lines and immortalized esophageal epithelial cell lines. EC171, EC9706, KYSE150, EC109 and KYSE510 were ESCC cell lines. NE1, NE2 and NEcA6 were immortalized esophageal epithelial cell lines. **(D)** Immunofluorescence analysis of ATF3 expression in KYSE150 cells, an ESCC cell lines with high-expression of ATF3 (400×). **(E)** Comparison for the invasive capability of cells lines with different ATF3 expression level.

We first examined the expression of ATF3 in the progression from normal epithelium to carcinoma of the esophagus by using immunohistochemical staining. ATF3 was positive-expression in all cases of normal squamous cell epithelium in a cytoplasm-staining pattern (100%, 21/21). It was absent in the basal layer and strongly positive in the intermediate and superficial layers. In simple hyperplasia (75%, 6/8), mild dysplasia and moderate dysplasia (70%, 7/10), ATF3 was also present in the intermediate and superficial layers, whereas in severe dysplasia (71.4%, 5/7), positive staining was only observed in the superficial layers (Figure 1A). Comparatively, ATF3expression was significantly decreased in ESCC samples, showing a positive-expression rate of 51.3% (77/150) (Supplementary Figure S1). In addition, decreased expression of ATF3 was also found in human ESCC tissues compared with the paired normal tissues from the patients as shown by Western blotting analysis (Figure 1B).

ATF3 expression in 5 ESCC cell lines and 3 immortalized esophageal epithelial cell lines was also determined by using Western blotting. Results showed that ATF3 expressed in a low level in most of ESCC cell lines evaluated whereas in a high level in the 3 immortalized esophageal epithelial cell lines (Figure 1C). Confocal scanning revealed that ATF3 was predominantly distributed in the cytoplasm of ESCC cells (Figure 1D). Moreover, the invasive capability of these cells was addressed by chamber invasiveness assay and a negative correlation was found between ATF3 expression and cell invasion (*r* = −0.77, Pearson's Correlation analysis, Figure 1E).

### Impact of ATF3 expression on OS and DFS in ESCC patients

To obtain a better understanding of the clinical significance of ATF3 expression, we correlated its expression in the cancerous tissues with a series of clinicopathological features. As shown in Supplementary Table S1, no significant associations were observed between ATF3 expression and the clinicopathological features indicated.

**Figure 2:**
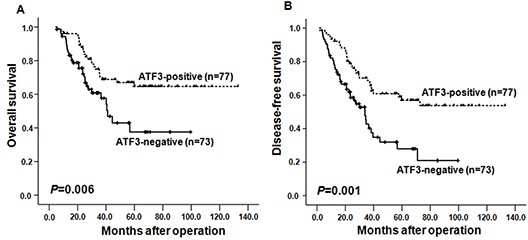
Kaplan-Meier curves depicting overall survival (OS, A) and the disease-free survival (DFS, B) according to expression patterns of ATF3 in ESCC samples The *P* values were calculated using the log rank test.

Kaplan-Meier survival analysis demonstrated that ATF3 positive expression predicted significantly better OS (*P*=0.006) and DFS (*P*=0.001) (Figure 2A and 2B). The median survival time of patients whose primary ESCC scored high for ATF3 expression was more than 80 months whereas negative ATF3 expression correlated with a shortened median survival time of about 35 months.

Supplementary Table S2 summarizes the results of the univariate analysis of prognostic variables. The following factors were significantly associated with the survival of patients besides ATF3 expression: regional lymph node metastasis (OS, *P*=0.002, DFS, *P*=0.001) and pTNM-stage (OS, *P*=0.047, DFS, *P*=0.02). Moreover, multivariate Cox regression analysis indicated that ATF3 expression was an independent positive prognostic factor for ESCC patients (Supplementary Table S2).

### Effect of ATF3 expression on cell growth and invasion *in vitro*

**Figure 3:**
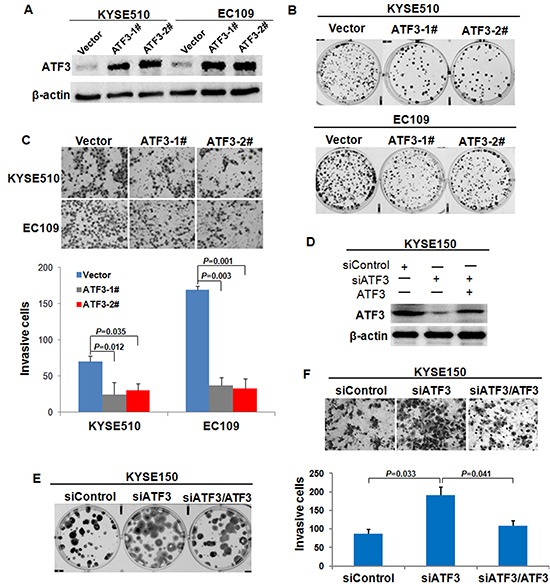
Effect of ATF3 expression on cell growth and invasion **(A)** Forced expression of ATF3 in EC109 and KYSE510 ESCC cell lines were addressed by Western blotting analysis. ATF3-1# and ATF3-2# were two different ATF3-transfected cell clones; Vector was cells transfected with vector control. **(B)** Colony formation assay was used to evaluate the growth of ATF3-expressing cells. **(C)** Invasiveness assay was used to determine the effect of ATF3 forced expression on cell invasion. Representative tumor cells invaded were photographed (400×), data represent mean ± SD of triplicates. **(D)** RNAi-mediated knockdown (siATF3) and re-expression of ATF3 (siATF3/ATF3) in KYSE150 cells were determined by Western blotting. Colony formation assay **(E)** and invasiveness assay **(F)** were employed to address the alterations of cell growth and invasion upon ATF3 knockdown and re-expression.

To assess the functional role of ATF3 expression in ESCC cells, we over-expressed ATF3 in EC109 and KYSE510 and then determined the effect exerted by forced expression of ATF3 on cell growth and invasion. Two clones in each cell line with high expression of ATF3 are shown in Figure 3A, which were used for further study. Colony formation assay, MTT assay and invasiveness assay revealed that ATF3 over-expression dramatically reduced cell growth and invasive properties in both EC109 cells and KYSE510 cells compared with control cells (Figure 3B and 3C, Supplementary Figure S2). The effect of ATF3 on cell growth and invasion was further confirmed in KYSE150 cells by using RNAi method. Results showed that with ATF3 knockdown, both of cell growth and invasion were promoted. Further analysis revealed that re-expression of ATF3 in the ATF3-silencing cells led to a 50% restoration of cell growth whereas resulted in a totally inversion of cell invasion (Figure 3D, 3E and 3F, Supplementary Figure S2). These findings suggested that effect of ATF3 on cell invasion was greater than that on cell growth, which prompted us to conduct further research on the molecular mechanisms of ATF3 role in cancer invasion.

### ATF3 over-expression suppressed the tumorigenesis and lung metastasis of ESCC cells *in vivo*

**Figure 4:**
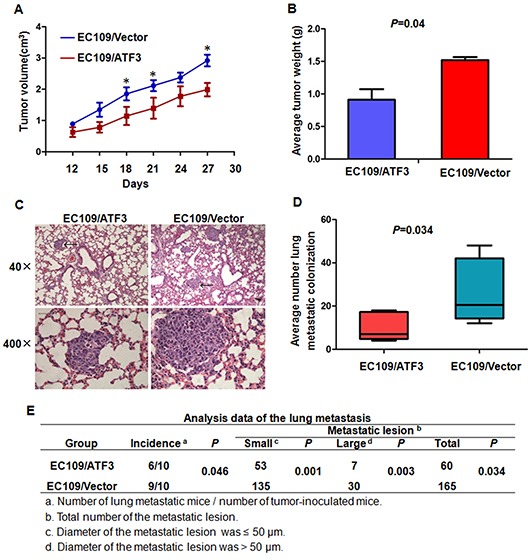
ATF3 expression suppressed cell growth and lung metastasis of ESCC cells in vivo **(A)** & **(B)** ATF3 forced expression EC109 cells and the control cells were implanted subcutaneously in nude mice. Tumor volume in different time points **(A)** and average weight of the tumors **(B)** were analyzed. **(C)** & **(D)** & **(E)** ATF3 forced expression EC109 cells and the control cells were inoculated via tail veins of the SCID mice. **(C)** The photomicrographs of H&E-stained lung tissues. Representative fields were shown and metastatic colonizations were marked with arrows. **(D)** and **(E)** Quantitative analysis of the number of surface lung metastasis colonization. *, *P* < 0.05.

To determine whether ATF3 plays an important role in the tumorigenesis and metastasis of ESCC cells, we injected ATF3-transfected EC109 cells or the control cells into the subcutis of nude mice or via tail veins of SCID mice. Consistent with the effect of ATF3 expression on the growth and invasion of ESCC cells *in vitro*, ATF3 over-expression significantly inhibited tumor growth (Figure 4A and 4B, Supplementary Figure S3). Moreover, in the metastasis model, cancer cells spreading into lung of mice was shown by H&E-staining. Number and size of the metastatic colonization were dramatically decreased on the lung surface of ATF3-transfected cells implanted mice compared to the control mice (Figure 4C, 4D and 4E). These data suggested that ATF3 inhibited tumorigenesis and metastasis of ESCC cells *in vivo*.

### Protein expression of MMP-2 was inhibited in the ATF3 over-expression cells

**Figure 5:**
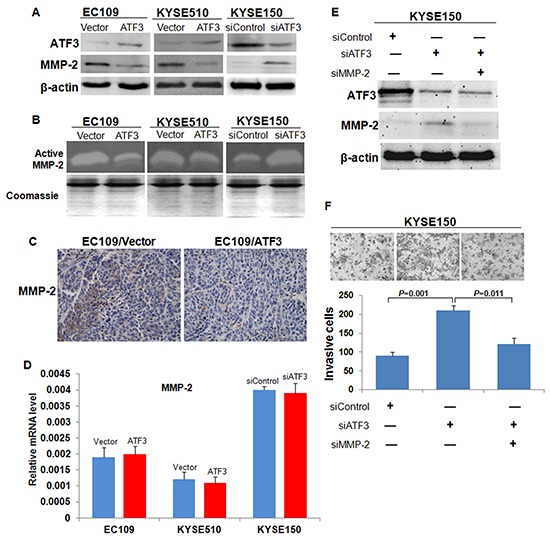
Expression and activity of MMP-2 in the ATF3 altered-expression ESCC cells **(A)** Western blotting analysis of MMP-2 expression in ATF3 forced expression or knockdown cells. β-actin served as a loading control. **(B)** Zymography of the conditioned medium for the activity of MMP-2. The coomassie staining of total protein in conditioned media were used to demonstrate that equal numbers of cells were present during the conditioning of the media. **(C)** Immunohistochemical staining of MMP-2 in the subcutaneous tumor tissues. Scale bar, 50μm. **(D)** Transcriptional level of MMP-2 was addressed by real time RT-PCR. **(E)** SiRNA targeted ATF3 (siATF3) and siRNA targeted MMP-2 (siMMP-2) were co-transfected into KYSE150 cells. **(F)** MMP-2 silencing inversed the increased cell invasion mediated by ATF3 knockdown.

We then investigated the mechanisms by which ATF3 modulated cancer cell invasion and metastasis. As a transcription factor, ATF3 might affect cell biological behaviors by regulating the expression of certain target genes. Hence, the expressions of several potential molecules downstream of ATF3 including Cyclin D1, β-catenin, MMP-2 and MMP-9 [7], were addressed by using Western blotting. Results showed that the protein levels of MMP-2 and β-catenin were increased with ATF3 over-expression (Supplementary Figure S4, Figure 5A). Based on the established role of MMP-2 in cancer metastasis, we selected MMP-2 for further study. Effect of ATF3 on MMP-2 expression was confirmed in KYSE510 cells and KYSE150 cells (Figure 5A). Zymography analysis of the conditioned media obtained from cells showed that the activity of MMP-2 was significantly lower in ATF3 over-expression cells and higher in ATF3 siRNA-treated cells, respectively (Figure 5B). Immunohistochemical analysis on the subcutaneous tumor tissues also confirmed that MMP-2 was decreased upon ATF3 expression (Figure 5C). However, results of real-time RT-PCR showed that the mRNA level of MMP-2 did not change with the alteration of ATF3 expression, suggesting that ATF3 did not regulate MMP-2 expression in transcriptional level (Figure 5D). To explore the role of MMP-2 in the ATF3-mediated cellular function, siRNA targeted ATF3 and siRNA targeted MMP-2 were co-transfected into KYSE150 cells. Results of invasiveness assay demonstrated that MMP-2 knockdown could inversed the increased invasion capability of ESCC cells resulted from ATF3 silencing, indicating that MMP-2 might be a major effector of ATF3-mediated suppression of cell invasion (Figure 5E and 5F).

### ATF3 mediated the degradation of MMP-2 in a MDM2 dependent manner

**Figure 6:**
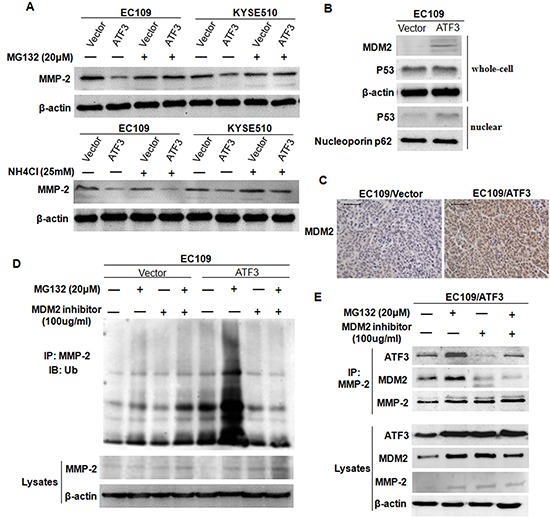
ATF3 mediated the degradation of MMP-2 in a MDM2 dependent manner **(A)** Transfected EC109 cells and KYSE510 cells were treated with MG132 or NH_4_Cl, and then harvested for Western blotting analysis of MMP-2. **(B)** Expressions of MDM2, total P53 and nuclear P53 in ATF3 forced expression EC109 cells was addressed by Western blotting. β-actin and Nucleoporin p62 served as loading controls. **(C)** Increased expression of MDM2 was confirmed in the subcutaneous tumor tissues by immunohistochemical staining. Scale bar, 50μm. **(D)** ATF3 forced expression EC109 cells and the control cells were treated with MG132, MDM2 inhibitor or both MDM2 inhibitor and MG132 and then used for co-immunoprecipitation. The ubiquitinated and the total level of MMP-2 were addressed by Western blotting. **(E)** The reciprocal protein complexes involving ATF3, MDM2 and MMP-2 in the ATF3-expressing cells. An antibody against MMP-2 was subjected to co-IP experiment and expression of ATF3, MDM2 or MMP-2 was addressed. β-actin was served as a loading control.

Next, to explore whether the ATF3-mediated reduced-expression of MMP-2 reflected altered MMP-2 protein stability, we treated the cells with MG132 (proteosome inhibitor) and NH_4_Cl (lysosome inhibitor) and the expression of MMP-2 in these cell was addressed. Results showed that protein level of MMP-2 was recovered in ATF3 over-expression cells when treated with MG132 but did not change when treated with NH_4_Cl, indicating that ATF3 might affect the degradation of MMP-2 through proteosome pathway (Figure 6A).

Previous studies have shown that ATF3 increased P53 stability and nuclear translocation and sequentially activated the transcription of mouse double minute 2 (MDM2), an E3 ubiquitin ligase [17, 18]. We therefore detected the expression of MDM2 in our cell models. Both Western blotting analysis on cell lysates and Immunohistochemical staining on the subcutaneous tumor tissues revealed that MDM2 were up-regulated with ATF3 forced expression (Figure 6B and 6C). In addition, nuclear expression of P53 was increased while the total P53 was unchanged in the ATF3 expressing cells, demonstrating that ATF3 may up-regulate MDM2 via P53 (Figure 6B). Further analysis by employing co-immunoprecipitation (co-IP) showed that the ubiquitination of MMP-2 was increased in MG132-treated cells, but when treated with MDM2 inhibitor or with both MDM2 inhibitor and MG132, the ubiquitination of MMP-2 was abolished and the protein level of MMP-2 was restored (Figure 6D). These findings suggested that ATF3 might mediate the degradation of MMP-2 in a MDM2 dependent manner.

To analyze the relationship among ATF3, MDM2 and MMP-2, an antibody against MMP-2 was subjected to co-IP experiment. Results revealed that the reciprocal protein complexes involving ATF3, MDM2 and MMP-2 could be detected in the ATF3 over-expression cells. Treatment of the cells with MG132 resulted in increased formation of the complex whereas treatment with MDM2 inhibitor did the opposite, confirming the existence of ATF3/MDM2/MMP-2 complex (Figure 6E). Moreover, we also found that ATF3 expression was regulated by MDM2-mediated proteosome pathway which was consistent with other studies (Figure 6E) [18].

### Cisplatin suppressed the invasion of ESCC cells by targeting ATF3

**Figure 7:**
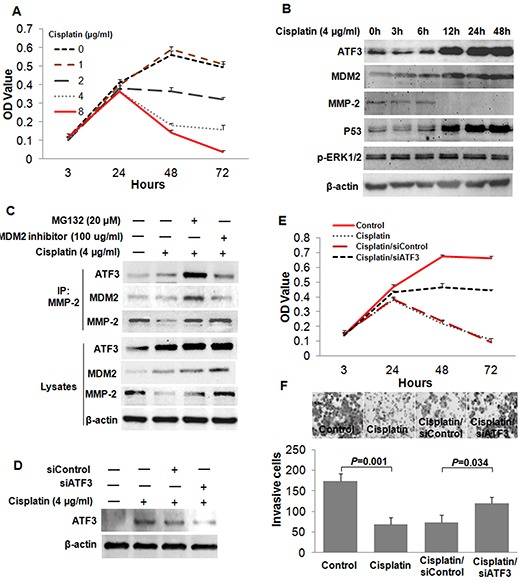
Cisplatin suppressed the invasion of ESCC cells by inducing the expression of ATF3 **(A)** Cytotoxicity of Cisplatin treatment on EC109 cells was determined by MTT assay. **(B)** Time course analysis for the expressions of ATF3, MDM2, MMP-2, P53 and p-ERK1/2 in EC109 cells treated with Cisplatin (4μg/ml). **(C)** Effect of Cisplatin on the formation of the ATF3/MDM2/MMP-2 complex. **(D)** ATF3 was silenced by RNAi method in the Cisplatin-treated cells. **(E)** MTT assay was employed to determine the effect of ATF3 silencing on Cisplatin-induced inhibition of cell growth. **(F)** Role of ATF3 in the Cisplatin-induced inhibition of cell invasion was addressed by invasiveness assay.

Finally, to determine whether the ATF3 was a target of cancer therapy, we treated the ESCC cells with Cisplatin, which is the most active antitumor agent used in human chemotherapy. The cytotoxic effect of Cisplatin on EC109 cells was analyzed by using MTT assay. Higher doses of Cisplatin exhibited obvious cytotoxicity to EC109 cells after treatment for 48 h and 72 h (Figure 7A). Then, a time-course experiment was conducted to gain insight into the kinetics of ATF3 protein expression upon Cisplatin treatment (4μg/ml). Samples were collected at six time points (0, 3, 6, 12, 24, 48 h) and significant ATF3 induction was found after 12 h of treatment. With the treatment of Cisplatin, MDM2 was up-regulated and MMP-2 was down-regulated (Figure 7B). P53 and ERK/MAPK pathways were previously reported to be involved in the Cisplatin-induced expression of ATF3 [19]. We found here that the activity of ERK/MAPK was unchanged while P53 expression was increased upon Cisplatin treatment, suggesting that Cisplatin might induce ATF3 via P53 (Figure 7B, Supplementary Figure S5). Results of Co-IP assay showed that, when treated with both MG132 and Cisplatin, formation of ATF3/MDM2/MMP-2 complex was increased, whereas MDM2 inhibitor treatment impeded the complex formation (Figure 7C). Moreover, invasiveness assay and MTT assay revealed that Cisplatin inhibited the invasion and growth of ESCC cells, and ATF3 knockdown was able to partially reverse this inhibition (Figure 7D, 7E and 7F). These data indicated that Cisplatin might suppress the invasion of ESCC cells by inducing the expression of ATF3.

## DISCUSSION

**Figure 8:**
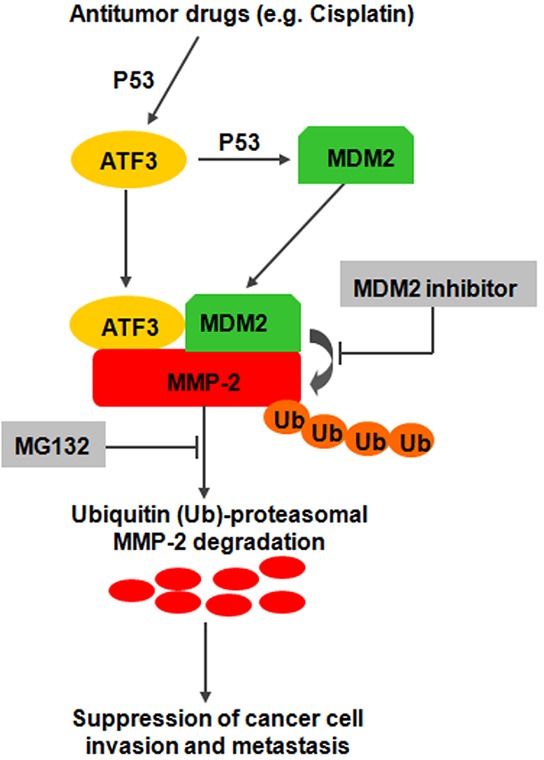
Proposed model illustrating opposing regulatory influences of ATF3 on MMP-2 degradation and cancer cell invasion and metastasis

In this study, we discovered four lines of evidence supporting a critical role for ATF3 in ESCC progression. First, we found a down-regulated ATF3 expression in ESCC, which was significantly associated with both OS and DFS of ESCC patients. ATF3 might serve as an independent prognostic factor. Second, forced expression of ATF3 led to decreased cell growth and invasive properties *in vitro* and *in vivo*, whereas knockdown of ATF3 did the opposite. Third, ATF3 suppressed the expression of MMP-2 by mediating the degradation of MMP-2 via proteosome pathway in a MDM2 dependent manner. Fourth, Cisplatin could suppress the invasion of ESCC cells by inducing the expression of ATF3 via P53 signaling. Therefore, we propose a new model for ATF3 as a novel suppressor of tumor invasion and metastasis in ESCC (Figure 8).

*ATF3* gene is located on human chromosome 1q32 within a region that is found to be frequently amplified in solid tumors [20]. In ESCC, a previous study showed that in the 29 cell lines that were analyzed, only one cell line (KYSE150) presented ATF3 amplification [21]. Hence, whether the *ATF3* gene is amplified or not in ESCC, especially in tissues needs further exploration. Herein, we firstly showed the reduced expression of ATF3 protein in ESCC and further revealed that ATF3 low-expression was associated with decreased survival. To our knowledge, this is the first report on the effect of ATF3 on the prognosis of ESCC patients. ATF3 has been demonstrated to be down-regulated in several types of tumors such as colon cancer and ovarian cancer [10, 11]. However, in prostate cancer and Hodgkin's lymphoma, ATF3 was found to be over-expressed and act as an oncogene, indicating that ATF3 may play differing roles in cancer development depending on the cell type [9, 22]. Much still remains to be learned about the complex roles of ATF3 in the context of tumor progression.

Invasion and metastasis has been shown to be one of the most important hallmarks of cancer [23]. ATF3 was discovered to be involved in cell invasion and metastasis of human ovarian cancer cells, lung cancer, and bladder cancer [11–13]. In current study, we showed that ATF3 expression decreased the invasive potential and suppressed the lung metastasis of ESCC cells, suggesting ATF3 might play as a metastasis suppressor. Moreover, lines of evidence suggest that ATF3 is essential for cell cycle progression. It was reported that ATF3 over-expression resulted in increased apoptosis of PC3 human prostate cancer cells, reduced focus formation, and reduced size of subcutaneous HCT-116 human colorectal cancer cell xenografts in nude mice [24, 25]. Consistently, the present study also showed over-expression of ATF3 suppressed the tumor growth *in vitro* and *in vivo* while ATF3 silencing did the opposite, supporting an important role for ATF3 in cell proliferation. Intriguingly, we found that effect of ATF3 on cell invasion was greater than that on cell growth, which prompted us to focus our studies on ATF3 role in cancer invasion.

Abnormal expression of ATF3 may mediate multiple aspects of cancer biology by repressing the transcription of certain downstream molecules including Cyclin D1, Id1 and IRS2 [7]. Our results suggested that the protein level and activity of MMP-2, a Type IV collagenase which was critical in cancer cell invasion and metastasis [26], was attenuated in the ATF3-expressing cells, suggesting that MMP-2 might be a downstream molecule of ATF3 function. Systemic metastases arise from tumor cells that disseminate via the blood and lymphatic vasculature. MMPs were shown to be essential for blood vessel invasion, but not lymphatic vessel invasion [27–29]. Here, we found that there was no correlation between ATF3 expression and lymph node metastasis in the analysis with clinical samples, whereas *in vivo* experiments showed that ATF3 expression was associated with lung metastasis. We proposed that ATF3 might inhibit the blood vessel invasion of ESCC cells by affecting the expression of MMP-2.

Previous study on human fibrosarcoma cells showed that ATF3 repressed MMP-2 expression by decreasing the transactivation of this gene [30]. However, we reported here that ATF3 suppressed the expression of MMP-2 by inducing the MDM2-mediated degradation, indicating that regulation of ATF3 on MMP-2 might have different mechanism in cells from different histological origin. Rapid degradation of specific proteins by ubiquitin/proteasome-dependent pathways is a component of many cellular regulatory mechanisms and emerging evidences suggested that degradation of protein plays important role in the progression of cancer [31]. MDM2, an E3 ubiquitin ligase, is known as oncogene for the ability to ubiquitylate P53 and lead to its proteasomal degradation [32]. However, it is believed that MDM2 has tumor suppressor functions by inducing cell cycle arrest or targeting Slug, a key transcriptional regulator in the EMT progression for proteolytic degradation [33, 34]. We found that ATF3 could increase the nuclear translocation of P53, and sequentially upregulate the expression of MDM2. Then, the formation of a complex including ATF3, MDM2 and MMP-2 was increased and thus facilitated MMP-2 degradation. All these findings indicated that ATF3 might inhibit the invasion and metastasis of ESCC cells by inducing the degradation of MMP-2. Study on the precise mechanism of the ATF3/MDM2/MMP-2 complex is underway.

Cisplatin is a clinically highly relevant anticancer drug used for the treatment of esophageal cancer. The known mechanism underlying its antitumor activity has been ascribed to the induction of DNA damage by platinum compounds and defining the specific mechanism(s) responsible for the antitumor affects of Cisplatin may lead to novel and improved therapeutic approaches [35]. Cisplatin was also reported to activate ATF3 through MAPK pathways or P53 signaling and ATF3 was important for Cisplatin's cytotoxicity [36]. Here, we confirmed the Cisplatin-induced expression of ATF3 via P53 and further showed that Cisplatin might suppress the invasion of ESCC cells by targeting the ATF3/MDM2/MMP-2 complex, suggesting that ATF3 is a potential treatment target in the chemotherapy of ESCC.

## MATERIALS AND METHODS

### Tissue specimens and immunohistochemical staining

Surgically removed tumors embedded in paraffin wax blocks from 150 ESCC cases, 21 paired adjacent normal epithelial tissues, 8 simple hyperplasia cases, 10 mild dysplasia or moderate dysplasia cases and 7 severe dysplasia cases were retrieved from the archives of the Department of Pathology of the Central Hospital of Shantou City, P.R. China. The cases were received between 1987 and 1999. The cases were selected in this study only if a follow-up was obtained and clinical data were available. The follow-up for patients after esophageal resection was continued until their deaths and only patients died from ESCC were included in the tumor-related deaths. The patients, suffering from severe post-operative complications, other tumors or died of other causes were excluded. For the use of these clinical materials for research purposes, we obtained prior consent from all patients and the study was approved by the ethical committee of the Central Hospital of Shantou City and the ethical committee of the Medical College of Shantou University.

The clinicopathologic data are summarized in Supplementary Table S3. Patients were staged according to AJCC Cancer Staging Manual for esophageal Carcinoma, 6^th^ Edition, 2002. All specimens were fixed in 10% formaldehyde solution, embedded in paraffin blocks, and selected to build tissue microarrays as described [37].

Immunohistochemical (IHC) staining was performed as described elsewhere [37]. The SuperPicTure Polymer Detection kit and the Liquid Substrate kit (Invitrogen, Carlsbad, CA) were used to carry out immunohistochemistry according to the manufacturer's instructions. Rabbit anti-human ATF3 polyclonal antibody (Rockland, Pennsylvania, USA) and Mouse anti-human ATF3 polyclonal antibody (ABGENT, San Diego, USA) were used. Each section was independently assessed by two histopathologists without prior knowledge of patient data.

Cases were scored based on immunostaining intensity (0, no staining; 1, weak staining; 2, moderate staining; 3, strong staining) and the percentage of positive cells (0, <5%; 1, 5%–25%; 2, 26%–50%; 3, 51%–75%; and 4, >75%). The final score of homogeneous staining was achieved by multiplication of the two scores above. For heterogeneous staining, each component was scored independently, and the results were summed. Using this system, the maximum score was 12. Scores 0–4 was categorized as “negative” and scores 5–12 as “positive” for the statistical analysis.

### Cell cultures

ESCC cell line EC171 and three immortalized esophageal epithelial cell lines (NE1, NE2 and NEcA6) were provided by professor Sai-Wah Tsao (Department of Anatomy, University of Hong Kong, China). Other four ESCC cell lines, EC109, KYSE150, EC9706 and KYSE510 were provided by professor Ming-Zhou Guo (Department of Gastroenterology & Hepatology, Chinese PLA General Hospital, Beijing, China). The immortalized esophageal epithelial cell lines were cultured in a 1:1 mixture of defined keratinocyte serum-free medium (Invitrogen) and EpiLife (Cascade Biologics, Portland, Oregon). The ESCC cells were cultured in DMEM medium (Invitrogen) containing 10% fetal calf serum. All cells were incubated at 37°C in a humidified atmosphere of 5% CO2 in air.

### Primers, siRNA and transfection

To generate the ATF3 expression vector, the open reading frame (ORF) of human ATF3 cDNA was cloned into the eukaryotic expression vector pcDNA3.0 (Invitrogen). For siRNA-mediated knockdown experiment, siRNA targeted 3'-UTR of *ATF3* gene (siATF3), siRNA targeted ORF of *MMP-2* gene (siMMP-2) and negative control siRNA (siControl) were purchased from QIAGEN (Germantown, MD, USA). For real time RT-PCR of MMP-2, the following primers were used: 5'-AGATCTTCTTCTTCAAGGACCGGTT-3' (forward) and: 5'-GGCTGGTCAGTGGCTTGGGGTA-3' (reverse).

The plasmid or siRNA was transfected into ESCC cells using HiPerFect reagent (QIAGEN). For the restored-expression of ATF3, siATF3 and ATF3 expression plasmid were co-transfected into KYSE150 cells. The transfected cells were harvested 48 h later and used for further analysis. In animal experiment, stable transfected clones were used. Briefly, G418 (400 μg/ml, Calbiochem, Darmstadt, Germany) was added to the culture medium 24 h after transfection. Stable G418-resistant clones were obtained in 7–9 days. The expanded cells were then used for *in vivo* studies.

### Western blotting and co-immunoprecipitation

Whole-cell lysates and nuclear protein were prepared from ESCC cells as described previously [38]. Standard Western blotting analysis of the lysates was performed with antibodies against ATF3 (Rockland), β-actin (Sigma, St. Louis, USA), MMP-2 (Thermo, San Jose, CA, USA), MDM2 (Santa Cruz, CA, USA), P53 (Cell Signaling Technology, Shanghai, China), p-ERK1/2 (Santa Cruz), or Ub (Santa Cruz). For co-immunoprecipitation (co-IP) studies, cells were grown in 10-cm-diameter tissue culture dishes. After treatment of MG132 (Calbiochem), MDM inhibitor (Santa Cruz) or Cisplatin (Hanson, Jiangshu, China), cells were lysed and the resulting supernatants were incubated on a rocker with 1 μg MMP-2 antibody for 1 h and then with 20 μl protein A/G PLUS Agarose (Santa Cruz) overnight at 4°C. The immunoprecipitates were collected and then subjected to SDS/PAGE analysis.

### Confocal laser scanning microscopy

The staining procedure was performed as described [39]. After fixation in 4% paraformaldehyde solution, cells were incubated with donkey serum blocking buffer and a primary antibody, followed by donkey anti-rabbit IgG (DyLight 488) (Jackson ImmunoResearch, West Grove, PA, USA). Samples were counter-stained with DAPI (Sigma) and the cells were finally examined under a confocal microscope (OLYMPUS, FV-1000).

### Cell growth study

The colony formation assay and MTT assay were used to evaluate cell growth. For MTT assay, cells were seeded in 96-well plates (5 × 10^3^ cells/well), and after incubation for 24 h, 48 h, 72 h or 96 h, MTT solution (5 mg/ml, Sigma) was added to the medium. The formazan crystals that formed were dissolved, and absorption was measured at 490 nm with an automatic ELISA reader. In the colony formation assay, cells were plated at a density of 200 cells/well in 6-well plates. They were then moved to a cell incubator. After 10 days, the number of colony-forming cells (>50 cells) was calculated under a microscope. The data were expressed as mean ± SD.

### Gelatin zymography

Cells were washed and cultured in serum-free 199 medium. After 24 h, the conditioned medium from 10^7^ cells was collected, concentrated 50-fold using a Nanosep 10K centrifugal device (Pall Corporation, Washington, USA), and subjected to SDS–PAGE through 10% polyacrylamide gels copolymerized with 1 mg/ml gelatin (Sigma). Gels were incubated overnight at 37°C and then stained with 0.1% Coomassie blue R250. After destaining, gelatinolytic signals were photographed. The coomassie staining of total protein in conditioned media were used to demonstrate that equal numbers of cells were present during the conditioning of the media.

### Cell invasiveness assay

Invasiveness assay was performed as described before [39]. Briefly, 1 × 10^5^ cells were seeded onto the top chamber of a 24-well Matrigel-coated membrane with 8-μm pores (BD Biosciences, New Jersey, USA), and the bottom chamber was filled with medium containing 10% fetal calf serum. The membranes were fixed and stained by Giemsa reagent 24 h later and invasive cells were quantified by counting 10 random fields under a light microscope (400×). The mean value was calculated from data obtained from three separate chambers.

### Tumorigenesis and experimental lung metastasis experiments *in vivo*

For tumorigenesis experiments, 1 × 10^6^ ATF3 forced-expression EC109 cells or control cells were injected subcutaneously into the right flank of the nude mice (male, 6 weeks old, 12 in each group). After 30 days of observation, the mice were sacrificed and the tumors were removed and weighed. In experimental lung metastasis experiments, two age-matched groups of SCID mice (male, 6 weeks old, 10 in each group) were injected with the indicated cells (1.2 × 10^7^) in 0.2 ml serum-free medium via tail veins. These mice were kept 28 days until they were sacrificed. Lungs of the SCID mice were removed and stored in 4% paraformaldehyde for examination by hematoxylin-eosin (H&E) staining.

All these mice were raised in specified pathogen-free conditions (26°C, 70% relative humidity, and a 12-h light/dark cycle). The use of animals complied with the Guide for the Care and Use of Laboratory Animals (NIH publication no. 86-23, revised 1985) and current Chinese law on the protection of animals.

### Statistical analysis

Comparisons between data sets were performed using the χ^2^ test or the Mann-Whitney U test. Overall survival (OS) was measured from the date of surgery to death from any cause. Disease-free survival (DFS) was measured from the date of surgery to disease progression or relapse. Probabilities of OS and DFS were calculated by the Kaplan-Meier method, and compared using the log-rank test. Relative risk was evaluated by the multivariate COX proportional hazards model. A two-tailed *P* value less than 0.05 was considered to have statistical significance. All statistical tests were performed with SPSS statistic software (SPSS® 14.0 by SPSS Inc).

## SUPPLEMENTARY FIGURES AND TABLES


