# Supplementary material for: ATF3 functions as a novel tumor suppressor with prognostic significance in esophageal squamous cell carcinoma
Source: Oncotarget. 2014 Aug 13;5(18):8569–82. doi: 10.18632/oncotarget.2322 (PMC4226705; doi:10.18632/oncotarget.2322)
Supplement: Supplementary file 1 [file oncotarget-05-8569-s001.pdf]

SUPPLEMENTARY FIGURES AND TABLES

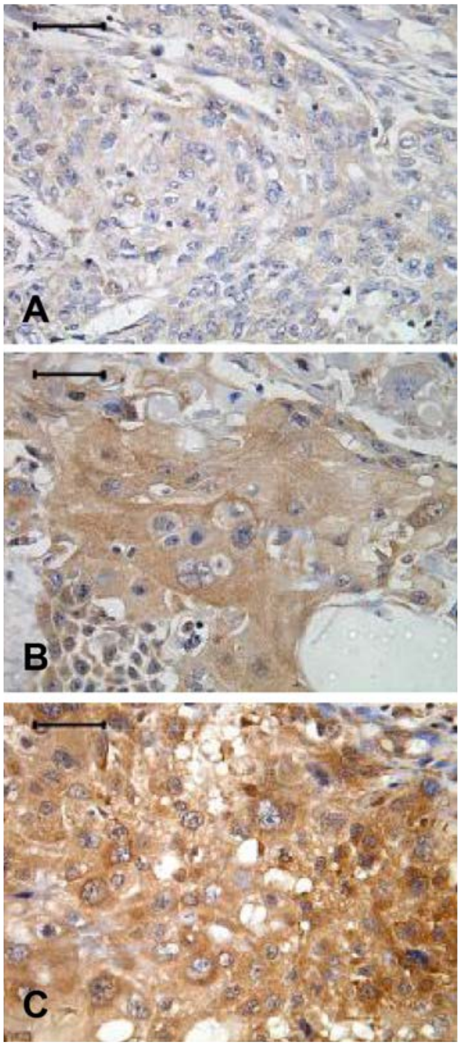

Supplementary Figure S1: Expression pattern of ATF3 in ESCC samples was confirmed by an ATF3 antibody purchased from ABGENT (at1222a). Cases with weak (A), moderate (B) or high expression (C) of ATF3 were shown. Bar, 50μm.

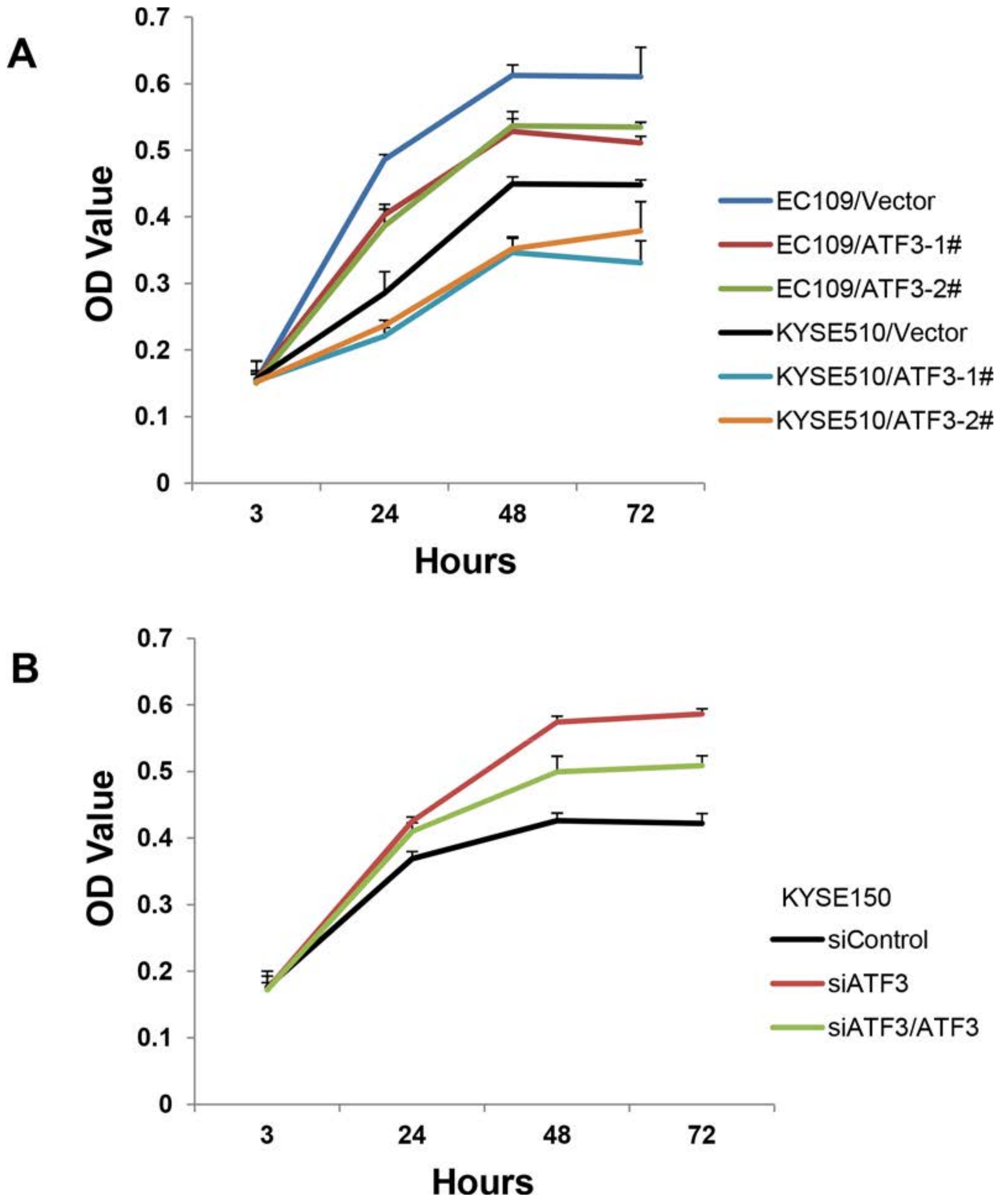

Supplementary Figure S2: ATF3 expression suppressed cell of ESCC cells *in vitro*. MTT assay was used to evaluate the growth of ATF3 forced-expression EC109 and KYSE510 cells (A), RNAi-mediated ATF3 knockdown KYSE150 cells (siATF3) and ATF3 re-expression KYSE150 cells (siATF3/ATF3) (B).

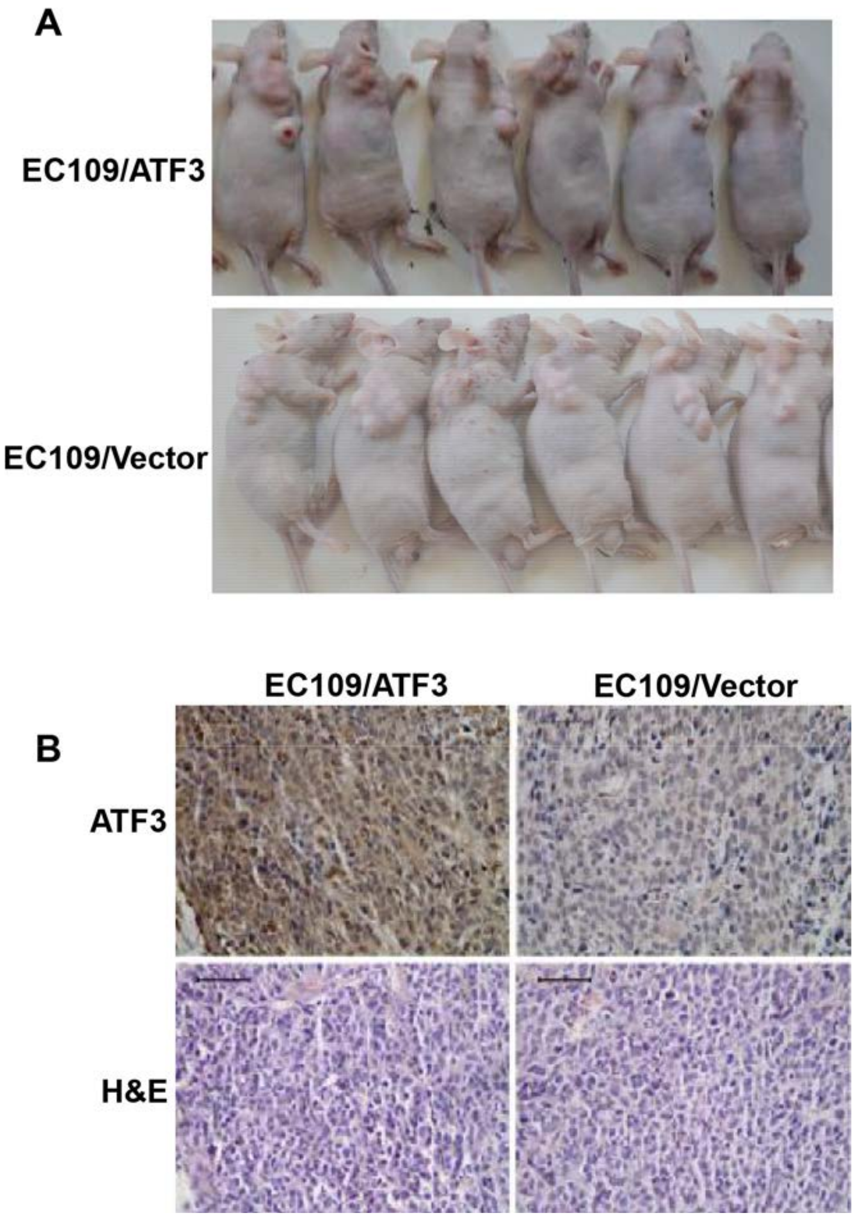

Supplementary Figure S3: Tumor formation in nude mice. **(A)** Representative pictures of the tumor bearing mice. **(B)** Immunohistochemical staining of ATF3 in the subcutaneous tumor tissues. Scale bar, 50μm.

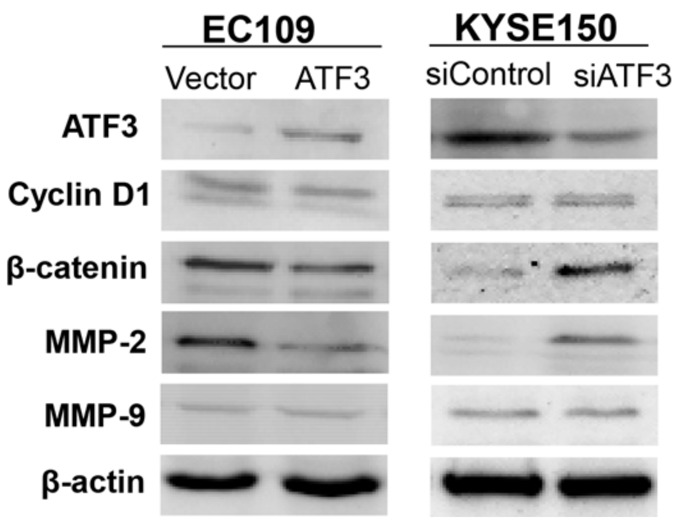

Supplementary Figure S4: Expressions of several potential molecules downstream of ATF3 in the ATF3-overexpressing EC109 cells or ATF3 knockdown KYSE150 cells. β-actin served as loading control.

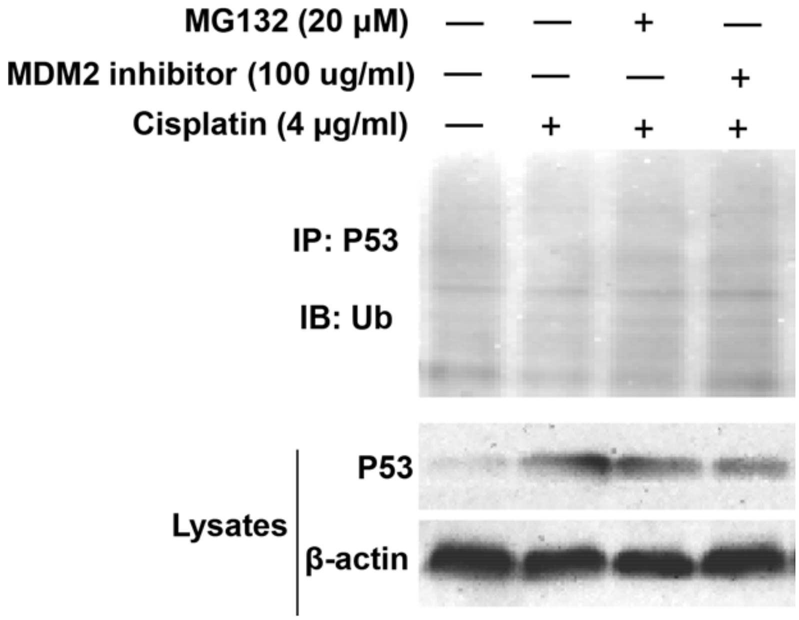

Supplementary Figure S5: Cisplatin did not effect the ubiquitination of P53 EC109 cells were treated with MG132, MDM2 inhibitor or/and Cisplatin and then used for co-immunoprecipitation by a P53 antibody. The ubiquitinated and the total level of P53 were addressed by Western blotting.

Supplementary Table S1. Correlation of clinicopathological features with protein expression levels of ATF3

Clinical Parameters	ATF3 expression		*P*
	Negative (%)	Positive (%)	
Age (year)			
<55	39(53.4)	44(57.1)	0.473†
≥55	34(46.6)	33(42.9)	
Gender			
Male	56(76.7)	55(71.4)	0.577*
Female	17(23.3)	22(28.6)	
Blood type			
A	16(21.9)	26(33.8)	0.085*
B	23(31.5)	11(14.3)	
AB	5(6.8)	4(5.2)	
O	29(39.7)	36(46.8)	
Tumor size			
≤3cm	18(24.7)	20(26.0)	0.167*
3–5cm	37(50.7)	49(63.6)	
>5cm	18(24.7)	8(10.4)	
Differentiation			
Well	21(28.8)	21(27.3)	0.374*
Moderate	37(50.7)	49(63.6)	
Poor	15(20.5)	7(9.1)	
Invasive depth			
T1+T2	13(17.8)	14(18.2)	1.000*
T3+T4	60(82.2)	63(81.8)	
Lymph node metastasis			
0	47(64.4)	51(66.2)	0.865*
1/2/3	26(35.5)	52(34.7)	
pTNM stage			
IA/IB/IIA/IIB	47(64.4)	53(68.8)	0.606*
IIIA/IIIB/IIIC/IV	26(35.6)	24(31.2)	

†Mann-Whitney U test; *χ^2 test

Supplementary Table S2. Univariate analyses and Multivariate analysis of factors associated with overall survival and disease-free survival

Variables	[a]OS		[b]DFS	
	HR (95%CI)	*P**	HR (95%CI)	*P**
**Univariate analysis**				
Age (≥55 vs <55)	0.754(0.435 to 1.306)	0.309	1.002(0.631 to 1.591)	0.994
Gender(Female vs Male)	1.378(0.727 to 2.615)	0.312	1.139(0.669 to 1.940)	0.631
Length of tumor		0.604		0.112
3–5cm vs ≤3cm	0.652(0.282 to 1.505)	0.316	0.454(0.217 to 0.952)	0.037
>5cm vs ≤3cm	0.794(0.401 to 1.572)	0.508	0.734(0.419 to 1.286)	0.279
Differentiation		0.068		0.059
G2 vs G1	0.387(0.174 to 0.864)	0.020	0.430(0.215 to 0.863)	0.018
G3 vs G1	0.605(0.310 to 1.179)	0.140	0.646(0.357 to 1.167)	0.148
Invasive depth (T4 vs T1/T2/T3)	0.772(0.407 to 1.464)	0.439	0.909(0.507 to 1.628)	0.750
Lymph node metastasis (N1 vs N0)	2.287(1.345 to 3.889)	0.002	2.262(1.423 to 3.595)	0.001
pTNM-stage (III/IV vs I/II)	1.492(0.869 to 2.560)	0.047	1.760(1.105 to 2.803)	0.020
ATF3 (positive vs negative)	0.478(0.278to 0.822)	0.006	0.432(0.269 to 0.694)	0.001
**Multivariate analysis**				
ATF3 (positive vs negative)	0.377(0.213 to 0.668)	0.001	0.379(0.233 to 0.618)	0.000

*Multivariate analysis, Cox proportional hazards regression model. Variables were adopted for their prognostic significance by univariate analysis.
[a]OS, Five-year over survival rate;
[b]DFS, Five-year disease-free survival rate;
Each *P* value is 2 tailed, and the significance level is 0.05.

Supplementary Table S3. Clinicopathlogical characteristics of patients

Clinical Parameters	No.	Survival	
		[a]OS	[b]DFS
Specimens	150		
Age (year)			
<55	83	49.3	44.7
≥55	67	61.5	45.0
Gender			
Male	111	51.9	44.5
Female	39	60.9	45.5
Blood type			
A	42	51.9	44.2
B	34	57.4	48.6
AB	9	33.3	23.7
O	65	56.2	46.3
Tumor size			
≤3cm	38	64.8	62.4
3–5cm	86	52.7	43.1
>5cm	26	43.9	30.0
Differentiation			
Well	42	63.8	54.9
Moderate	86	54.1	43.7
Poor	22	37.2	30.0
Invasive depth			
T1+T2	27	55.6	45.9
T3+T4	123	47.6	39.4
Lymph node metastasis			
0	98	64.5	56.2
1/2/3	52	33.8	27.4
pTNM stage			
IA/IB/IIA/IIB	100	59.5	52.0
IIIA/IIIB/IIIC/IV	50	42.9	30.9

[a]OS, Five-year over survival rate; [b]DFS, Five-year disease-free survival rate
